# Face concerns as identity-based social evaluation: a cultural psychological model of fertility intentions among Chinese women

**DOI:** 10.3389/fpsyg.2026.1844552

**Published:** 2026-06-12

**Authors:** Yuan Zhu, Zhirong Tian

**Affiliations:** 1 School of Humanities, Arts and Design, Guangxi University of Science and Technology, Liuzhou, China; 2 School of Economics and Management, Guangxi University of Science and Technology, Liuzhou, China

**Keywords:** birth rate, fertility intention, mianzi, structural equation modeling, women psychology

## Abstract

**Introduction:**

China’s declining fertility rate has raised significant demographic concerns. While prior research has largely emphasized economic and structural determinants, less attention has been paid to culturally embedded psychological mechanisms. This study examines how *mianzi* (face concerns) relates to Chinese women’s fertility intentions by integrating the Theory of Planned Behavior and Social Identity Theory.

**Methods:**

Survey data were collected from 446 Chinese women of reproductive age. *Mianzi* was conceptualized as a multidimensional construct comprising self-, relational-, and societal-oriented dimensions. Structural equation modeling was used to test the hypothesized relationships, including mediation and moderation effects.

**Results:**

All three *mianzi* dimensions were positively associated with attitudes toward childbearing. Relational- and societal-mianzi were also positively associated with subjective norms, whereas self-mianzi was not. Both attitudes and subjective norms were positively associated with fertility intention, with subjective norms showing a stronger association. Perceived behavioral control significantly moderated these relationships.

**Discussion:**

The findings suggest that fertility intentions are shaped not only by structural conditions but also by culturally embedded identity and social evaluation processes. The study highlights the importance of incorporating culturally sensitive psychological mechanisms into fertility research and policy discussions.

## 1 Introduction

In recent years, China has faced challenges in increasing its fertility rate, which has important implications for social stability and economic growth (Fan, 2026; Huang et al., 2025). The declining birth rate is not merely a demographic issue but a reflection of deeper societal and cultural shifts. Historically, Chinese society placed immense importance on family continuity and the bearing of children, particularly male heirs, as a means of preserving the family line (Chen et al., 2025). However, the rapid urbanization, increasing educational attainment, and rising participation of women in the workforce have led to changes in attitudes toward marriage and childbearing (Liu et al., 2025). Many Chinese women, particularly in urban areas, are prioritizing career development and personal fulfillment over traditional family expectations. Therefore, many Chinese women are choosing to delay or forgo childbirth, raising questions about the underlying factors influencing their reproductive choices (Hu et al., 2024).

While economic considerations, career aspirations, and the availability of childcare resources are frequently emphasized as key determinants of fertility intention, cultural pressures may operate in parallel by shaping what is viewed as socially appropriate or respectable for women (Karabchuk et al., 2022). Specifically, societal and family expectations may intensify pressure on women to align with traditional family roles, including motherhood, thereby generating tension between individual aspirations and culturally prescribed norms (Defu, 2022). In this sense, mianzi operates not merely as an interpersonal concern but as a cultural mechanism through which social approval, family reputation, and gender norms are reinforced.

In contemporary Chinese society, mianzi represents a socially grounded sense of dignity that is related to how individuals are evaluated and acknowledged by others. It is inherently interpersonal and context-dependent, linking personal dignity to public recognition and normative conformity within family and society (Zeng, 2024). Rather than being an internal trait alone, mianzi fundamentally relational and reflects one’s perceived standing within a network of social relationships (Buckley et al., 2006; Li, 2021). Maintaining mianzi involves safeguarding one’s reputation, respectability, and symbolic prestige in everyday interactions. As such, individuals are motivated not only to cultivate harmonious relationships but also to avoid behaviors that could result in social embarrassment or loss of respect (Lin, 2011). In this sense. mianzi plays a central role in shaping interpersonal dynamics and choices as a form of social currency. By demonstrating sensitivity to others’ mianzi through respect, compliance with social expectations, and reciprocal consideration, individuals are able to strengthen social ties and gain access to collective resources (Buckley et al., 2006). Preserving one’s own mianzi while protecting that of others facilitates trust, reinforces reciprocity, and sustains long-term relational networks in Chinese society (Zhou and Zhang, 2024). Accordingly, mianzi is particularly relevant for understanding individual decision-making in the Chinese context.

Previous research on fertility decisions has largely focused on economic and policy-driven factors, such as income levels, employment opportunities, and access to childcare (Buyukkececi et al., 2020; Emokpae and Brown, 2021; Raybould and Sear, 2021). Scholars have explored the impact of government policies like the two-child policy and its effects on fertility rates (Wang and Sun, 2020).

In addition, more recent research has increasingly expanded the understanding of fertility intentions by incorporating emerging social, technological, and contextual determinants beyond traditional economic and demographic factors. For instance, recent longitudinal evidence such as Lu et al. (2025a) highlights the association between technological change and shaping fertility behavior. In parallel, growing attention has been paid to social and peer-related influences on women’s fertility decisions. Contemporary studies such as Lu et al. (2025b) emphasize that fertility decisions are increasingly negotiated within contexts of social comparison and perceived life-course expectations.

In the realm of cultural studies, mianzi has been explored extensively in relation to business practices, interpersonal relationships, and social behavior (Zhuo and Yuan, 2022). However, the gap in the literature lies in the nuanced understanding of how cultural pressures, particularly mianzi, intersect with modern social dynamics and link with Chinese women’s reproductive decisions. Addressing this gap is crucial for developing a holistic understanding of fertility trends in China, which can inform both policy and social initiatives aimed at addressing the country’s demographic challenges.

In this light, this study addresses the following research question: How does mianzi relate to Chinese women’s fertility intention? To answer this question, we conceptualize mianzi as a multi-dimensional construct comprising three dimensions, namely, self-mianzi, relational-Mianzi, and societal-mianzi, to reflect the multi-layered nature of face culture in China. This multi-dimensional framework is appropriate because mianzi functions simultaneously at the intrapersonal level (self-evaluation and dignity), the interpersonal level (family obligations and relational harmony), and the societal level (public reputation and compliance with wider social expectations). Specifically, self-mianzi captures individuals’ internalized sense of dignity and alignment with personal ideals (Zhou and Zhang, 2017). Relational-mianzi reflects face concerns embedded in close relationships, emphasizing the fulfillment of family expectations and the maintenance of familial harmony (Defu, 2022). Societal-mianzi represents the pressure to conform to broader societal norms and to maintain one’s public reputation and social legitimacy (Zhou and Zhang, 2017).

The theoretical foundation of this study integrates the Theory of Planned Behavior (TPB) and Social Identity Theory (SIT). TPB explains how attitudes and subjective norms relate to behavioral intentions (Conner, 2020), while SIT highlights the role of group membership and social identity in motivating behavior (Stets and Burke, 2000). Building on these perspectives, this study conceptualizes mianzi as the independent variable and examines its association with Chinese women’s intention to give birth. Specifically, we propose that mianzi positively relates to women’s intentions through key TPB mechanisms. However, the associator could be different in different demographic settings. To empirically test the proposed model, data were collected through a structured survey of Chinese women of reproductive age, and the hypothesized relationships were examined using structural equation modelling with control variables tested.

By integrating cultural perspectives, this research contributes to a more comprehensive understanding of fertility trends in China. It underscores the need for culturally sensitive policies that address not only the economic and logistical barriers to childbearing, but also the underlying cultural values that relate to reproductive decisions. The findings provide practical insights for policymakers and relevant stakeholders seeking to understand women’s psychology and respond to China’s fertility challenges.

## 2 Theoretical framework and hypotheses development

### 2.1 Theoretical foundation

This study uses the Theory of Planned Behavior (TPB) as the primary theory and Social Identity Theory (SIT) as the supplementary theory to explain how mianzi relates to Chinese women’s intention to give birth. TPB argues that behavioral intention is jointly determined by attitude toward the behavior, subjective norms, and perceived behavioral control (Ajzen, 1991). SIT further emphasizes that individuals’ behavioral choices are related to social identity and group-based norms, as individuals seek to maintain a positive identity and avoid disapproval from valued reference groups (Ashforth and Mael, 1989; Islam, 2014).

At its core, TPB posits that behavior is driven by behavioral intentions, which in turn are determined by three interrelated constructs: attitudes toward the behavior, subjective norms, and perceived behavioral control (Conner and Sparks, 2015). Each of these constructs provides a distinct but interdependent pathway through which cultural and social influences relate to decisions (Smith, 2015).

In the context of Chinese women’s reproductive decisions, *attitudes* are deeply intertwined with traditional values emphasizing family continuity, social harmony, and self-fulfillment through motherhood (Nie et al., 2023). *Subjective norms*, another pillar of TPB, refer to the perceived social pressures to perform or abstain from a given behavior (Conner and Sparks, 2015). In a collectivist society like China, these norms are amplified by mianzi. A woman’s decision to have children is not only a personal choice but also a reflection of familial expectations and societal values (Hertz, 2006). The pervasive influence of subjective norms underlines the necessity to conform to culturally sanctioned roles to maintain or enhance one’s mianzi*. Perceived behavioral control*, the final component of TPB, pertains to an individual’s perceived ease or difficulty in performing a behavior, reflecting both external barriers and internal capacities (Hertz, 2006). For Chinese women, this includes managing the financial, professional, and psychological challenges associated with motherhood (Kadale et al., 2018).

Within TPB, subjective norms are typically operationalized as injunctive normative beliefs, reflecting individuals’ perceptions of whether important referents approve or disapprove of a given behavior (Ajzen and Klobas, 2013). In fertility research, such norms commonly capture perceived expectations from significant others, such as partners, parents, or close friends regarding childbearing decisions (Ajzen and Klobas, 2013). Hence, TPB-based models primarily emphasize normative pressure in terms of social approval or disapproval.

By contrast, mianzi is not reducible to perceived social expectations. Rooted in Confucian relational ethics, mianzi represents a culturally embedded system of identity evaluation, reputational accountability, and social legitimacy. It encompasses concerns related to family honor, moral standing, and the preservation of socially recognized dignity, extending beyond immediate behavioral expectations to the maintenance of a valued social identity over time (Filieri et al., 2017).

Unlike subjective norms, which refer to perceived pressure from significant others regarding whether one should perform a specific behavior, mianzi operates through identity-based concerns about how one’s actions affirm or threaten socially recognized worth (Li et al., 2026). In this sense, subjective norms capture behavior-specific expectations, whereas mianzi captures the identity and reputational consequences of meeting or violating those expectations. As a result, two individuals may perceive similar subjective norms regarding fertility, yet differ substantially in how strongly those expectations are internalized as identity-relevant, leading to divergent attitudes and intentions.

Accordingly, in the theoretical model, mianzi is conceptualized as a cultural–identity orientation that shapes how individuals interpret the meaning of childbearing in relation to dignity, family legitimacy, and social recognition. In contrast, attitudes and subjective norms are fertility-specific psychological evaluations that directly precede intention formation within the TPB (Zhang et al., 2026). Thus, mianzi does not directly translate into fertility intention; rather, it conditions the interpretive lens through which fertility-related beliefs and expectations acquire psychological significance.

Based on this distinction, SIT is adopted to explain why mianzi operates as an identity-regulatory mechanism rather than as a simple form of social pressure. SIT posits that individuals derive part of their self-concept from social group memberships and are motivated to maintain a positive social identity because group-based evaluation is closely tied to self-worth and social standing (Trepte, 2006). This perspective is particularly appropriate for mianzi, which has been conceptualized as socially granted prestige contingent on recognition by relevant others and subject to gain, maintenance, or loss in interaction (Hu, 1944). Importantly, this does not imply mere compliance with group expectations, but rather reflects the motivation to preserve identity value within culturally meaningful evaluative systems.

Accordingly, this study conceptualizes mianzi as an identity-regulatory construct operating through three SIT-based processes: in-group identification, social evaluation, and identity maintenance. Relational-mianzi reflects in-group identification, as it captures the extent to which individuals’ sense of self is tied to maintaining legitimacy within close relational groups such as the family. This is not simply conformity to family expectations, but the preservation of relational identity and belonging. Societal-mianzi corresponds to social evaluation and comparison processes, reflecting concerns about how one is positioned within broader social hierarchies and public standards of respectability. Rather than representing generalized social pressure, it captures the reputational consequences of being evaluated by wider audiences. Self-mianzi represents identity maintenance, referring to internalized standards of dignity and self-worth that guide behavior to avoid identity threat. It reflects the regulation of behavior to preserve a coherent and socially valued self-concept.

By mapping these dimensions onto SIT mechanisms, the model extends beyond TPB by explaining not only how attitudes and subjective norms relate to intention, but also why these evaluations carry identity significance in culturally embedded contexts. In this framework, individuals do not simply respond to perceived expectations; they engage in behavior that protects or enhances their socially recognized identity.

By contrast, mianzi is not reducible to perceived social expectations. Rooted in Confucian relational ethics, mianzi represents a socially embedded system of self-evaluation, reputational responsibility, and identity maintenance, encompassing concerns about family honor, social recognition, moral standing, and the long-term consequences of social (Filieri et al., 2017). Unlike subjective norms, which operate through compliance with others’ expectations, mianzi functions through internalized anticipations of shame, loss, or damage to one’s self- and family image, thereby shaping individuals’ attitudes toward behavioral risks and benefits as well as their intentions to act in identity-consistent ways (Filieri et al., 2017). Consequently, two individuals may perceive similar subjective norms regarding a behavior, yet differ substantially in their sensitivity to mianzi, leading to divergent attitudes and intentions.

Therefore, in the theoretical model, mianzi is conceptualized as a relatively broad cultural-identity orientation that relates to how women interpret childbearing in relation to dignity, family reputation, and social legitimacy from inside to outside (Li et al., 2026). By contrast, attitudes and subjective norms are fertility-specific psychological evaluations that precede intention formation. Accordingly, mianzi is not expected to translate automatically or directly into fertility intention. To be more specific, subjective norms refer to perceived pressure from important others regarding a specific behavior, namely, whether significant referents think one should have a child. Relational- and societal-mianzi, however, refer to broader identity-based concerns about preserving family harmony, maintaining public respectability, and avoiding loss of face. Thus, mianzi reflects the cultural-identity lens through which social expectations become psychologically salient, whereas subjective norms capture the fertility-specific perception of such expectations. These mechanisms are consistent with TPB, in which attitudes and subjective norms are the most proximal antecedents of behavioral intention.

Based on the above discussions, SIT is adopted because mianzi is fundamentally an identity-evaluative, group-contingent social resource rather than a purely individual attitude. SIT argues that individuals derive part of the self-concept from social group memberships, and that they are motivated to maintain a positive social identity because group-based evaluation is closely tied to self-worth and social standing (Trepte, 2006). This lens is especially appropriate for mianzi research because classic work on “face” in Chinese societies conceptualizes mianzi as socially granted prestige that depends on recognition by relevant others and can be gained, maintained, or lost in interaction; it is therefore inherently relational and public, reflecting whether one is seen as a legitimate and respected member of valued social circles (Hu, 1944).

Therefore, this study conceptualizes mianzi as an identity-regulatory mechanism operating through three core processes: in-group identification, social evaluation/comparison, and identity maintenance. For instance, relational-mianzi reflects in-group identification, as it captures individuals’ sensitivity to expectations from close social groups such as family members (Defu, 2022). In line with SIT, individuals derive part of their self-concept from these groups and are motivated to align their behavior with group norms to maintain belonging and relational legitimacy. Societal-mianzi corresponds to social comparison and external evaluation, as it reflects concern with how one is perceived relative to broader social standards and reference groups (Defu, 2022). This dimension aligns with SIT’s emphasis on comparative evaluation processes through which individuals assess their social standing and seek positive distinctiveness. Self-mianzi captures internalized identity standards, reflecting identity maintenance processes whereby individuals regulate their behavior to preserve self-worth and avoid identity threat. Although less directly tied to external group pressure, this dimension reflects the internalization of socially constructed identity expectations.

By mapping these dimensions onto SIT mechanisms, the model extends beyond TPB by explaining not only how attitudes and norms influence intentions, but also why these evaluations are socially meaningful because they are tied to identity validation within culturally salient reference systems. Accordingly, SIT provides a clear theoretical bridge for explaining why mianzi concerns translate into behavioral intentions: individuals engage in norm-congruent choices and avoid norm-deviant choices to protect or enhance social identity value as evaluated by significant in-groups and reference groups. As a complementary theory, it contributes to the framework by positing that individuals derive a significant portion of their self-concept from membership in social groups and that they strive to maintain a positive social identity by conforming to group norms and expectations (Stets and Burke, 2000).

### 2.2 Theoretical hypothesis

**Figure 1:**
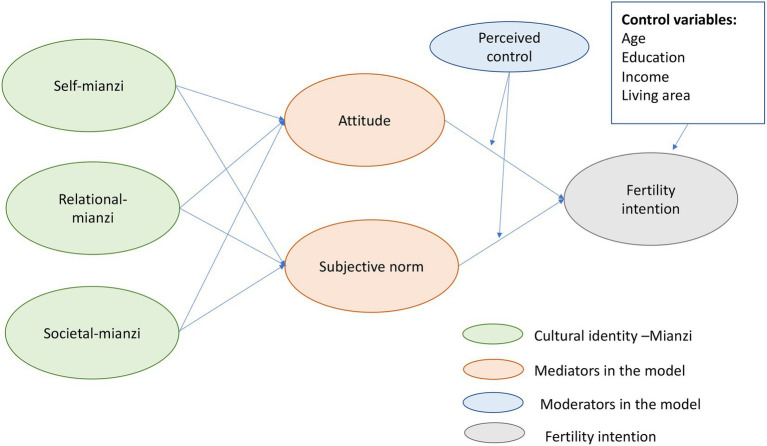
The theoretical model.

Figure 1 presents the proposed theoretical framework. In this study, mianzi (face culture) is conceptualized as a multidimensional cultural system consisting of three distinct but related dimensions: self-mianzi, relational-mianzi, and societal-mianzi. Each of which independently influences fertility-related attitudes and subjective norms. Rather than treating mianzi as a higher-order latent construct, we model these three dimensions as separate first-order cultural drivers, reflecting the possibility that different face concerns operate through distinct psychological and social mechanisms in fertility decision-making.

Specifically, self-mianzi captures concerns related to personal dignity, self-worth, and moral adequacy; relational-mianzi reflects sensitivity to expectations and evaluations from close social ties, particularly family members and elders; and societal-mianzi represents concern for public reputation, social recognition, and conformity to broader societal norms (Abrams and Hogg, 2001; Rosenberg, 2017; Shultziner and Rabinovici, 2012). Modeling these dimensions allows us to disentangle how different layers of face consciousness relate to women’s evaluative judgments and perceived social pressures surrounding childbearing.

In the context of fertility decision making, childbearing is widely regarded as a culturally meaningful and morally evaluated life-course choice. Concerns related to self-mianzi are therefore closely associated with how women evaluate childbearing, as fulfilling socially valued roles such as motherhood is often linked to personal dignity and self-respect, contributing to more favorable attitudes toward childbirth (H1a). At the same time, heightened self-mianzi is associated with stronger sensitivity to internalized standards of social evaluation, corresponding to higher perceived subjective norms surrounding fertility decisions (H1b).

Relational-mianzi, which centers on maintaining family harmony and meeting intergenerational expectations, is similarly associated with fertility-related evaluations and perceived social pressure. Women who place greater importance on relational face concerns are more likely to view childbearing positively as a means of fulfilling family obligations (H2a) and to perceive stronger normative expectations from significant others regarding fertility (H2b).

At the societal level, societal-mianzi reflects concern for public image and conformity to socially endorsed life trajectories. In social contexts where motherhood functions as a marker of social maturity and respectability, stronger societal face concerns are associated with more favorable attitudes toward childbearing (H3a) as well as heightened awareness of societal expectations regarding fertility (H3b).

Attitudes toward childbearing and subjective norms are positioned as the most immediate psychological correlates of fertility intention. Favorable evaluations of childbearing correspond to higher willingness to plan for childbirth (H4), while stronger perceived social expectations correspond to greater motivation to align intentions with culturally and socially prescribed roles (H5). TPB’s construct of perceived behavioral control highlights how external resources enable individuals to act on their intentions (Hagger et al., 2022; Zolait, 2014). Perceived support may amplify the influence of mianzi by reducing barriers to fulfilling its expectations. Together, these evaluative and normative considerations constitute central components in the formation of fertility intention in culturally embedded contexts.

Based on the above arguments, we propose the following hypotheses:

*H1a*: Self-mianzi has a positive association with attitudes

*H1b*: Self-mianzi has a positive association with subjective norms

*H2a*: Relational-mianzi has a positive association with attitudes

*H2b*: Relational-mianzi has a positive association with subjective norms

*H3a*: Societal-mianzi has a positive association with attitudes towards fertility

*H3b*: Societal -mianzi has a positive association with subjective norms

*H4*: Attitudes has a positive association with fertility intention

*H5*: Subjective norm has a positive association with fertility intention

*H6*: Perceived control has a moderation effect on fertility intention

## 3 Methodology

### 3.1 Research design

This study employs a cross-sectional survey design, collecting quantitative data to test the research hypotheses. The survey approach allows for the efficient collection of a large amount of data from a diverse sample of Chinese women of reproductive age. By using a structured questionnaire, we are able to assess various aspects of mianzi culture and personal attitudes, as well as the heterogeneity among demographic factors.

As latent constructs are involved, we use measurement items to operationalize the constructs. All measurement items were adapted from well-established and previously validated scales, with wording modifications made to ensure conceptual consistency with the women’s fertility and childbirth decision-making context. Specifically, the mianzi constructs (self-mianzi, relational mianzi, and societal mianzi) were adapted from prior face-consciousness and mianzi scales developed and validated in Chinese and East Asian cultural contexts (e.g., Chan et al., 2015; Zhang and Zhou, 2024; Zhao et al., 2022). The original items were reworded to explicitly reference childbirth, fertility, and family continuation, while preserving the original theoretical meaning of face as self-related dignity (self-mianzi), relational harmony and obligations (relational mianzi), and public reputation and social evaluation (societal mianzi). The attitude, subjective norms, perceived behavioral control, and fertility intention measures were adapted from established fertility and family-planning studies grounded in the Theory of Planned Behavior (e.g., Söderberg et al., 2013; Dommermuth et al., 2011; Matera et al., 2023; Yan et al., 2025). Item wording was adjusted to reflect the respondents’ personal fertility considerations without altering the underlying construct definitions. All items were measured using a 7-point Likert scale ranging from 1 (“strongly disagree”) to 7 (“strongly agree”) (see Table 1).

**Table 1:** Measurement items.

Constructs	Items	Reference
Self-mianzi	I find it difficult to admit doubts or concerns about having children because it affects my sense of personal dignity	Zhang and Zhou (2024)
	*I tend to avoid talking about personal vulnerabilities related to pregnancy or childbearing*	
Relational mianzi	I believe it is important to respect elders’ views regarding childbirth and family continuation.	Chan et al. (2015)
	I avoid confrontation with family members about childbirth expectations to maintain harmony	
Societal mianzi	Having children is important for maintaining social respect and recognition	Chan et al. (2015) and Zhao et al. (2022)
	I care about how others compare my life choices about childbirth with those of other women	
	Receiving social approval for fulfilling expectations related to childbirth is important to me.	
Attitude	Having a child is a way for me to add new elements in life	Söderberg et al. (2013)
	Having children is an essential part of life	
	It is important for me to be fertile	
Perceived behavioural control	I feel I would be capable to give birth	Ajzen (2002)
	I have control over the fertility choice	
Subjective norms	My partner wants me to have a child.	Dommermuth et al. (2011)
	My close friends want me to have a child	
	The important ones want me to have a child	
Fertility intention	I have the willingness to have atl east a child	Matera et al. (2023) and Yan et al. (2025)
	I am happy to have a child in the next few years	

As this study targets Chinese women, purposive sampling was adopted to collect data. This is because we exclude male and under-aged respondents. To enhance the representativeness of the sample, we cover a wide range of reproductive ages and experiences from different regions of China. Online surveys as distributed to approach a larger number of respondents. The survey was distributed with the assistance of a professional survey company, Credamo, from September 1st to September 30th 2024. We reviewed the response to exclude answers that (1) answered within 2 min (2) were incomplete (3) failed to pass the attention checker. This helps to enhance the reliability of responses. 368 surveys were collected. After the screening, a total of 345 valid responses were retained for analysis. Others were deleted due to the above-mentioned excluded reasons.

To improve sample size adequacy and enhance the robustness of the empirical analysis, data were collected in two rounds approximately 9 months apart. The second round was conducted to supplement the initial sample, as the first wave (September 2024) yielded a valid sample size (*N* = 345) that was sufficient but close to the lower bound recommended for structural equation modeling with multiple latent constructs. The additional data collection (June–July 2025) increased statistical power and improved the stability of parameter estimates.

To assess the comparability of the two samples, we conducted independent sample t-tests between the first-wave and second-wave respondents across key constructs and demographic variables. The results indicate no statistically significant differences, suggesting that the two samples can be treated as drawn from the same underlying population (Lahaut et al., 2003). Furthermore, following established procedures, early and late responses (corresponding to the two rounds) were compared to evaluate potential non-response bias, and no significant differences were observed.

Regarding potential contextual changes between the two data collection periods, we acknowledge that fertility-related discourse and policy environments in China may evolve over time. However, no major structural policy shifts directly affecting fertility behavior (e.g., new nationwide birth policies) were introduced during the interval between the two waves. Moreover, the core constructs examined in this study reflect relatively stable cultural and psychological orientations rather than short-term reactions to policy fluctuations. Therefore, the likelihood that temporal variation materially biases the consistency of responses is limited but still acknowledged.

### 3.2 Sample demographics

The final sample consists predominantly of young and early mid-life women. Specifically, 57.8% of respondents are aged 18–29 and 33.9% are aged 30–39, while women aged 40 and above account for 8.3% of the sample. This age distribution indicates that the sample is skewed toward women who have not yet entered, or are at an early stage of, family formation, a group for whom fertility intentions are particularly salient.

With respect to socioeconomic characteristics, respondents are distributed across income levels, with a concentration in the lower-to-middle income range. Approximately 68.0% report a monthly income below 10,000, while a smaller proportion earn above this level. Educational attainment is relatively high: over 72% of respondents hold at least a bachelor’s degree, including 22.6% with a master’s degree and 12.5% with a PhD or higher. This profile reflects a well-educated cohort for whom opportunity costs, career considerations, and identity-related concerns are especially relevant to fertility decision-making. Geographically, respondents are fairly evenly divided between northern (52.9%) and southern (47.1%) regions, suggesting reasonable regional coverage and reducing the likelihood that the findings are driven by a single regional context.

Overall, the sample is best understood as representing predominantly young, unmarried, and well-educated women who are largely in the intention-formation stage, rather than the realization stage, of fertility behavior. Accordingly, the findings should be interpreted as reflecting the cognitive, normative, and attitudinal processes through which women evaluate and anticipate future fertility, rather than actual childbearing outcomes. The final sample consists primarily of young and early mid-life women, with 57.8% aged 18–29 and 33.9% aged 30–39, while women aged 40 and above account for 8.3% of the sample, indicating that the sample is skewed toward women who have not yet entered, or are at an early stage of, family formation. With respect to socioeconomic characteristics, respondents are distributed across income levels, with a concentration in the middle-income range. Approximately 68.0% report a monthly income below 10,000, while a smaller proportion earn above this level. Educational attainment is relatively high: over 72% hold at least a bachelor’s degree, including 22.6% with a master’s degree and 12.5% with a PhD or higher, reflecting a well-educated cohort. Geographically, respondents are fairly evenly split between northern (52.9%) and southern (47.1%) regions, suggesting reasonable regional coverage.

Overall, this sample is best understood as representing predominantly young, unmarried, and well-educated women who are largely in the intention-formation stage rather than the realization stage of fertility behavior. Accordingl, the findings should therefore be interpreted as reflecting the decision-making context of women evaluating or anticipating future fertility (see Table 2).

**Table 2:** Demographic information.

Group	Item	Frequency	Percentage (%)
Age	18–29	285	57.8
	30–39	161	33.9
	40 and above	37	8.3
Income	Below 2000	53	11.9
	2001–4,000	73	16.4
	4,001–6,000	81	18.2
	6,001–1,000	96	21.5
	10,001–20,000	120	26.9
	20,001 and above	23	5.2
Education	High school	120	26.9
	Bachelor’s degree	169	37.8
	Master’s degree	101	22.6
	PhD and above	56	12.5
Living area	Northern area	205	52.9
	Southern area	241	47.1

### 3.3 Bias and reliability test

To assess common method bias, a common latent factor was introduced and connected to all observed items in the measurement model. The model was reanalyzed to extract standardized loadings. Differences between loadings with and without the common latent factor were computed. These differences were below 0.2, indicating that common method bias does not significantly affect this study (Archimi et al., 2018).

Potential concerns regarding reliability and collinearity among latent constructs were explicitly examined, as high correlations may bias structural path estimates and compromise interpretability in structural equation modeling.

To evaluate the reliability of the measurement scales, internal consistency was examined. The results indicate that all constructs meet commonly accepted reliability standards, with Cronbach’s alpha coefficients exceeding the 0.70 threshold. Specifically, Cronbach’s alpha values were 0.714 for self-mianzi, 0.721 for relational mianzi, and 0.848 for societal mianzi. Attitude (*α* = 0.919) and fertility intention (*α* = 0.909) exhibited excellent internal consistency, while subjective norms also showed satisfactory reliability (*α* = 0.729). In addition, all measurement items exhibited corrected item–total correlation (CITC) values above 0.50, suggesting that each item contributes adequately to its intended latent construct (Churchill and Iacobucci, 2006).

To assess potential multicollinearity among the predictors, variance inflation factors (VIFs) were examined. Multicollinearity reflects the degree to which independent variables are linearly related and may undermine estimation accuracy in regression models (Kreijns et al., 2022; Nanjundeswaraswamy et al., 2025). Consistent with prior recommendations, VIF values below 4 indicate an acceptable level of independence among constructs. As all VIF values in the model fall below this threshold, multicollinearity does not pose a concern for the present analysis.

## 4 Results

This section presents the findings from the data analysis, starting with the measurement model’s validation, followed by the structural model analysis and hypothesis testing. The results include confirmatory factor analysis for reliability and validity checks and structural equation modeling for hypothesis testing.

### 4.1 Measurement model validation

Before testing the structural model, we conducted a confirmatory factor analysis (CFA) to ensure the validity and reliability of the latent constructs. The CFA results indicated a good fit for the measurement model: CFI = 0.949, TLI = 0.933, RMESA = 0.074, SRMR = 0.066. The fit indices met the commonly accepted thresholds (CFI and TLI > 0.90; RMSEA < 0.10; SRMR < 0.08), indicating that the measurement model is a good fit for the data.

Other statistics are listed in Table 3. The composite reliability (CR) values for all constructs exceeded 0.70, indicating good internal consistency. The average variance extracted (AVE) for each construct was greater than 0.50, demonstrating adequate convergent validity. Discriminant validity was assessed by comparing the AVE values to the squared correlations between constructs. All AVE values were greater than the squared correlations, confirming discriminant validity (Fornell and Larcker, 1981).

**Table 3:** Confirmatory factor analysis results.

Constructs	Indicator	λ	AVE	CR
Self-mainzi (SEA)	SEA1	0.751	0.572	0.727
	SEA2	0.762		
Relational-mianzi (REM)	REM1	0.765	0.554	0.713
	RME2	0.724		
Society mianzi (SOM)	SOM1	0.807	0.727	0.841
	SOM2	0.896		
Subjective norms (SUN)	SUN1	0.772	0.606	0.754
	SUN2	0.785		
	SUN3	0.704		
Attitude for giving birth (ATT)	ATT1	0.935	0.830	0.965
	ATT2	0.927		
	ATT3	0.870		
Fertility intention (INT)	INT1	0.882	0.752	0.933
	INT2	0.858		
	INT3	0.863		

### 4.2 Model comparison

**Figure 2:**
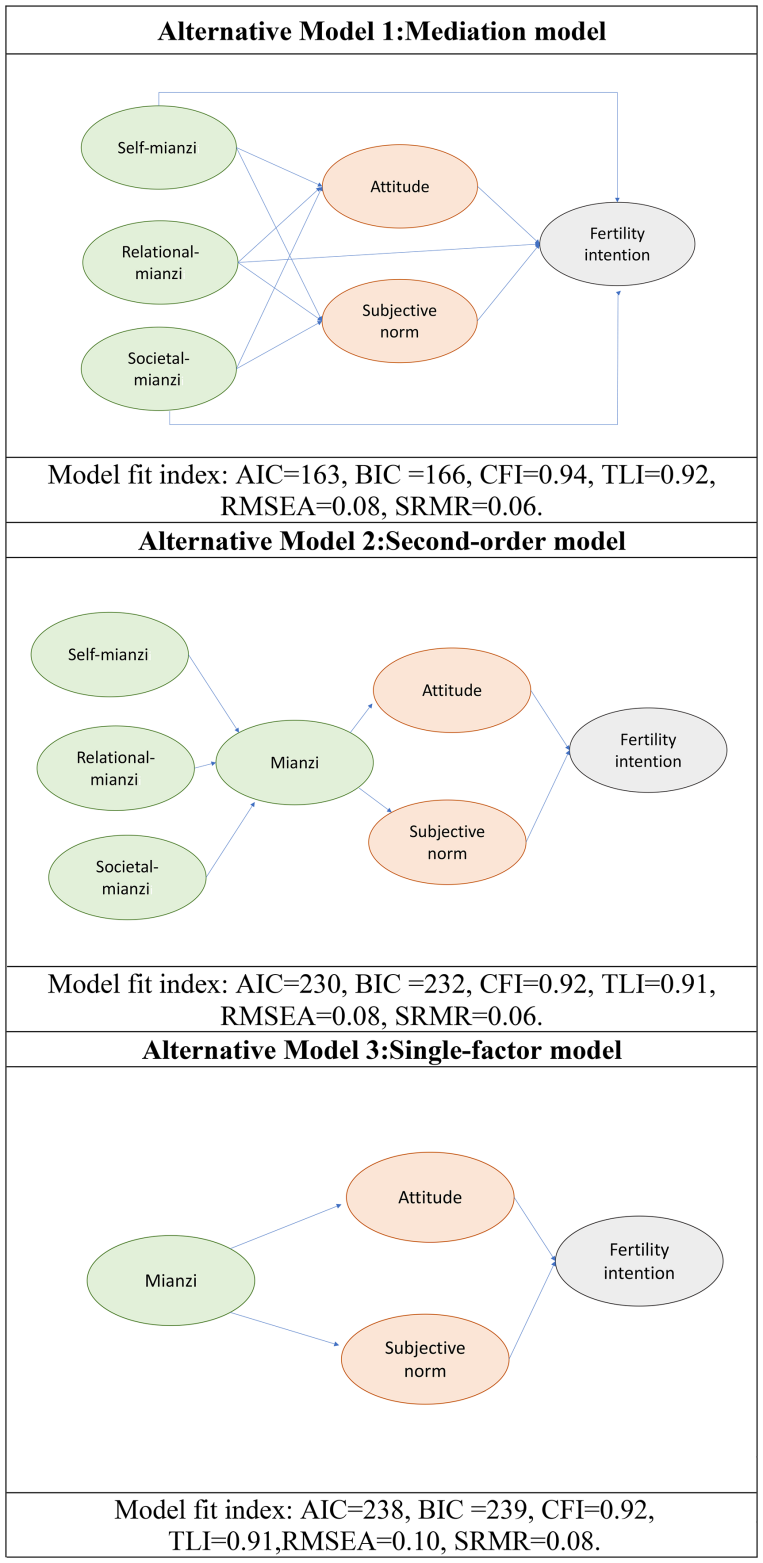
Alternative models.

To evaluate the suitability of the proposed research framework for testing the hypothesized relationships, several alternative models were developed based on the original model (Huang, 2023). First, a nested model (Alternative Model 1) was specified by adding additional direct paths. The results show that both the AIC and BIC values of this model are slightly higher than those of the original model (AIC = 59; BIC = 162). Moreover, among the three added paths, only one is statistically significant. In accordance with the principle of parsimony, the original model therefore represents a more appropriate specification. The original model was further compared with a second-order factor model (Alternative Model 2) and a one-factor model (Alternative Model 3). In both cases, the original model demonstrates superior model fit and provides a more nuanced explanation of the distinct dimensions of mianzi culture. Accordingly, these results indicate that the original full mediation model fits the data better than the alternative specifications, thereby supporting the hypothesized relationships (see Figure 2).

### 4.3 Structural model results

**Figure 3:**
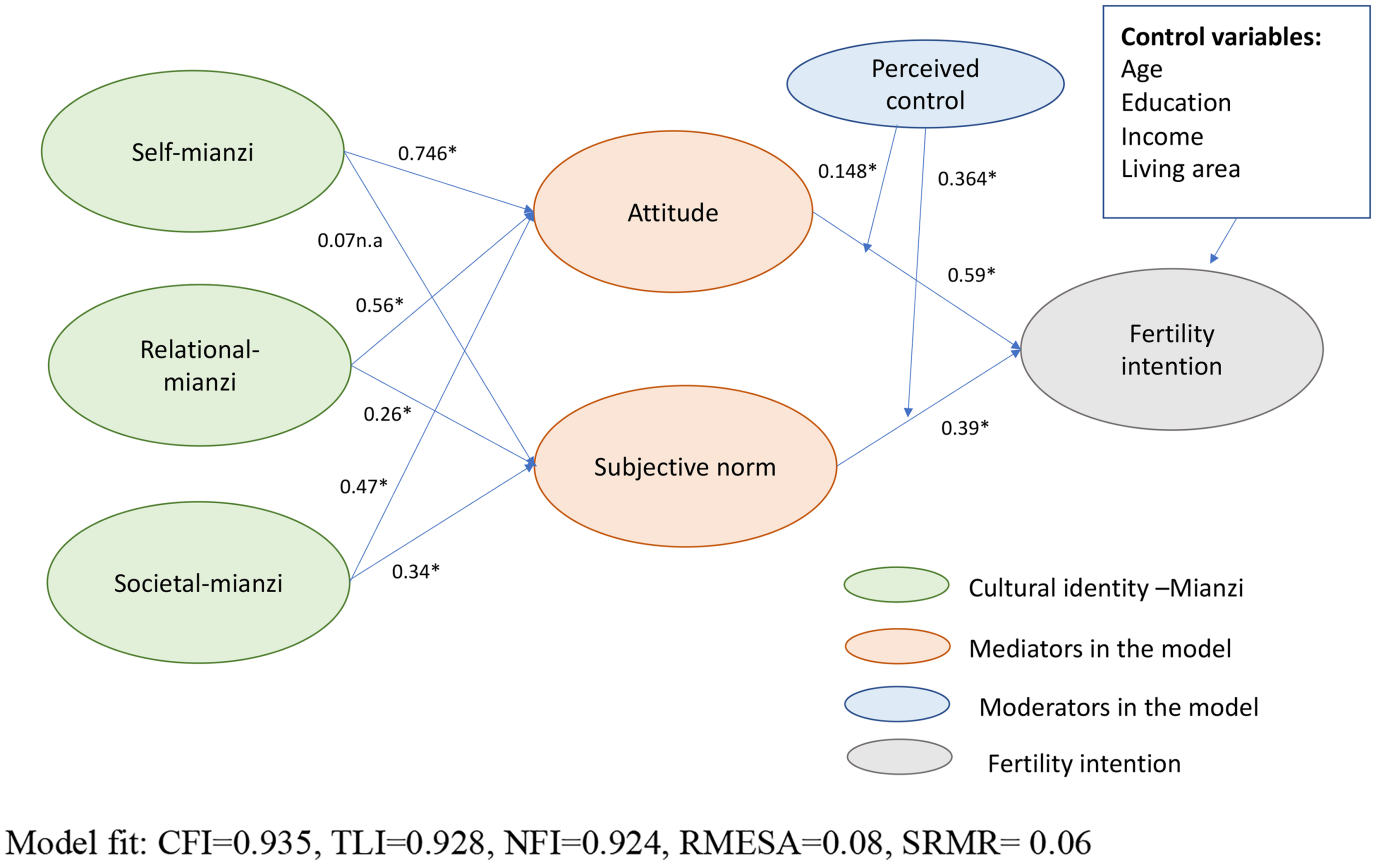
The estimated coefficient.

We control for age, education, living area, and income in the model for robustness across demographic segments. The standardized estimates are presented in Figure 3.

Regarding the control variables, age is found to be significantly and negatively associated with fertility intention (*b* = −0.086, *p* < 0.001). Education level shows a positive but non-significant association with fertility intention (*b* = 0.030). Living place is significantly and negatively related to fertility intention (*b* = −0.052, *p* < 0.05). Income level is positively associated with fertility intention, although this relationship does not reach statistical significance (*b* = 0.024).

After controlling for the demographic variables, these results indicate that three dimensions of mianzi have positive associations with attitudes, supporting H1a, H2a and H3a. Self-mianzi is not found to be significantly related to subjective norms, rejecting H1b. Relational-mianzi and societal-mianzi are found to be significantly related to subjective norm, supporting H2b and H3b. Attitude and subjective norm are positively related to fertility intention, supporting H4 and H5.

The testing of moderation effect of perceived control is based on the indicant product approach proposed by Ping (1995). The basic logic is to use cross-product of measurement items to examine if the interaction of latent variables has a significant effect on the exogenous variable. To illustrate, the indicators of perceived control, attitude, and subjective norm are mean-centred before performing cross-product to overcome multicollinearity issues. Thereafter, the measurement items of perceived control, attitude, and subjective norms are summed, respectively. The summed values of perceived control and attitudes, as well as perceived lack of value and subjective norms, are multiplied separately to create single indicators of the latent interaction factor. The significance of the interaction factor is tested subsequently by running the model. It is found that the interaction factor of perceived control and attitudes has a positive association (*b* = 0.148, *p* < 0.001) on fertility intention; the interaction factor of perceived control and attitudes has a positive association (*b* = 0.364, *p* < 0.001) on fertility intention. The results suggest that consumers who lack sufficient perceived control are more strongly associated with their attitudinal evaluations and normative pressures when forming fertility intentions. Specifically, the positive and significant interaction effects indicate that, under conditions of low perceived control, favorable attitudes toward childbearing and stronger subjective norms exert an amplified effect on fertility intention. This pattern implies that when individuals feel constrained in their ability to autonomously manage fertility-related decisions, they rely more heavily on internal evaluations and socially shared expectations as compensatory decision cues. Such findings underscore the contingent role of perceived control in shaping how attitudinal and normative factors translate into fertility intentions.

A bootstrapping procedure was employed to assess indirect effects in the mediation model. Specifically, 500 resamples were generated to approximate the sampling distribution of the indirect paths. Estimates of the direct, indirect, and total effects are reported in Table 4. The findings indicate attitude is a mediator between self-mianzi, relational-mianzi, societal-mianzi and fertility intention. Subjective norm is a mediator between relational-mianzi, societal-mianzi, and fertility intention.

**Table 4:** Mediation analysis results.

Exogenous (*i*)	Endogenous (*j*)		
	Subjective norm (1)	Attitude (2)	Fertility intention (3)
Direct effects (*a_ij_*) of (bootstrapping)			
Self mianzi (1)	0.07 [−0.03; 0.9]	0.74 [0.61; 0.8]	–
Relational mianzi (2)	0.26 [0.21; 0.31]	0.56 [0.46; 0.54]	–
Societal mianzi (3)	0.34 [0.28; 0.41]	0.47 [0.38; 0.56]	–
Subjective norm (4)	–	–	0.39 [0.33; 0.46]
Attitude (5)	–	–	0.59 [0.49; 0.71]
Indirect effects (*b_ij_*) of (bootstrapping)			
Self mianzi–attitude (1a)	–	–	0.43 [0.21; 0.44]
Self mianzi–norm (1b)			0.02 [−0.13; 0.03]
Relational mianzi–Attitude (2a)	–	–	0.33 [0.28; 0.36]
Relational mianzi–norm (2b)			0.10 [0.08; 1.30]
Societal mianzi–attitude (3a)	–	–	0.27 [0.22; 0.35]
Societal mianzi–norm (3b)			0.13
Total effects (*c_ij_*) of …			
Self mianzi (1)	0.07	0.74	0.45
Relational mianzi (2)	0.26	0.56	0.43
Societal mianzi (3)	0.34	0.47	0.40
Subjective norm (4)	–	-	0.39
Attitude (5)	–	–	0.59

### 4.4 Discussion of results

This study examines how different dimensions of mianzi are statistically associated with attitudes, subjective norms, and fertility intention within a TPB-based framework. The findings contribute to the growing literature that situates fertility decision-making within culturally embedded evaluative and normative contexts.

First, the observed association between all three dimensions of mianzi and attitudes toward childbearing is consistent with prior research emphasizing that fertility-related evaluations are shaped by culturally defined role expectations and identity meanings (Nie et al., 2023). In particular, the stronger association of self-mianzi with attitudes aligns with existing conceptualizations of mianzi as an internalized form of dignity and self-evaluation (Zhang, 2015). This suggests that fertility-related attitudes are not merely preference-based but are linked to identity-relevant interpretations of life-course choices. Such an interpretation is consistent with studies indicating that culturally valued roles, including motherhood, are embedded in evaluative frameworks tied to self-worth and social legitimacy (Yu and Liang, 2022; Zhou and Zhang, 2024).

Further, the differentiated association between mianzi dimensions and subjective norms refines existing understandings of social influence in fertility research. While prior TPB-based studies emphasize the role of perceived expectations from significant others (Manning, 2009), the present findings suggest that not all forms of cultural pressure operate uniformly. The stronger alignment of relational- and societal-mianzi with subjective norms is consistent with literature highlighting the importance of family expectations, social comparison, and public recognition in collectivist contexts. At the same time, the absence of a significant association between self-mianzi and subjective norms supports distinctions within the mianzi literature between internally oriented and externally oriented face concerns (Shi et al., 2017). This pattern indicates that perceived normative pressure is more closely associated with socially visible and relationally embedded forms of evaluation.

In addition, the association of both attitudes and subjective norms with fertility intention, with subjective norms showing a relatively stronger association, is consistent with prior TPB research in collectivist cultural settings (Kumar and Nayak, 2023). Attitudes serve as a psychological bridge between cultural influences and behavioral intentions (Zebregs et al., 2015). According to TPB, positive attitudes increase the likelihood of an individual forming a strong intention to act (Ranjbarian et al., 2012; Sentosa and Mat, 2012). In the context of childbearing, attitudes reflect a woman’s evaluation of motherhood as a meaningful and desirable role. These evaluations are not made in isolation but are deeply informed by cultural narratives and social pressures. For women who perceive childbearing positively, strong intentions are more likely to follow.

Finally, the limited role of structural variables such as income and education in the present model is consistent with recent studies suggesting that fertility intentions are not solely associated with socioeconomic position but are also linked to culturally embedded meanings and normative expectations. This pattern aligns with emerging literature that highlights the importance of psychological and social mechanisms in shaping fertility-related orientations, particularly in contexts where traditional norms and modern life-course trajectories coexist.

## 5 Conclusion

### 5.1 Theory contribution

This study received approval from the relevant institutional review board with the approval number YX20240720H008 to cover the research period from 01/08/2024 to 12/31/2026. Informed consent was obtained from all participants, who were informed of the study’s purpose, the voluntary nature of participation, and the anonymity of their responses. No personally identifiable information was collected, and all data were stored securely in accordance with established ethical guidelines for social science research and will not be shared to any third-party.

This study contributes to the literature in three key ways. First, it advances fertility research by introducing mianzi as a culturally embedded identity-based mechanism that relates to fertility intention. While prior studies have predominantly focused on structural, economic, and policy-related determinants, the present findings demonstrate that fertility decisions are also related to social evaluation and identity regulation processes.

Second, the study contributes to the Theory of Planned Behavior by showing that its core components can be understood as differentiated pathways through which cultural identity concerns operate. Specifically, self-mianzi is aligned with evaluative processes (attitudes), whereas relational and societal mianzi are aligned with normative processes (subjective norms). This provides a more fine-grained explanation of how culturally embedded factors are translated into TPB constructs.

Third, the study contributes to cultural psychology and Social Identity Theory by demonstrating that identity-based concerns are not uniform but multi-layered and functionally differentiated. By disaggregating mianzi into self-, relational-, and societal dimensions, the findings show how different forms of identity evaluation relates to decision-making through distinct psychological mechanisms.

The findings suggest that mianzi is not a unitary cultural trait but a differentiated system that aligns with distinct components of the TPB framework. Self-oriented mianzi is more closely aligned with evaluative judgments reflected in attitudes, whereas reputation-related and social mianzi are more closely aligned with perceived social expectations reflected in subjective norms. This differentiated pattern reinforces the value of disaggregating cultural constructs when examining fertility-related orientations and avoids conflating culturally embedded identity concerns with generalized social pressure. This patterned alignment provides empirical support for integrating Social Identity Theory with TPB by illustrating how culturally grounded identity concerns map onto evaluative versus normative belief structures without collapsing mianzi into generalized social pressure. More broadly, the results underscore the importance of theorizing cultural values as structured and differentiated systems that relates to behavioral orientations through multiple psychological pathways, rather than treating culture as a uniform or static factor in fertility decision-making.

## 6 Practical implications

The empirical findings indicate that attitudes toward childbearing are closely aligned with multiple dimensions of mianzi, with the strongest alignment observed for self-mianzi and additional significant alignment for relational- and societal-mianzi. A culturally specific implication is that public communication and institutional messaging may be more effective when they engage with the symbolic and identity-related meaning of childbearing, rather than focusing solely on material incentives. In particular, communication strategies may emphasize the compatibility of childbearing with personal dignity, self-development, and socially respected life trajectories (self-mianzi), while also highlighting socially valued themes such as preparedness, responsibility, and relational stability (relational and societal mianzi). Such framing aligns with the evaluative component of TPB by shaping how childbearing is interpreted within culturally embedded standards of self-worth and social recognition.

The results further indicate that subjective norms are more strongly aligned with relational- and societal-mianzi than with self-mianzi, suggesting that perceived social expectations surrounding fertility are primarily embedded in interpersonal and public-facing forms of social evaluation. Accordingly, norm-oriented approaches may benefit from focusing on reference-group dynamics and socially shared narratives, rather than individual-level persuasion alone. For example, community-based communication, digital platforms, and local organizations may highlight narratives that present supportive parenting arrangements as socially recognized and “face-preserving.” At the same time, normative messaging may shift emphasis away from evaluative standards such as “having a child as a marker of worth” toward more process-oriented recognition, such as “establishing a stable and supportive family context,” thereby remaining consistent with mianzi-related concerns without reinforcing social pressure.

In addition, both attitudes and subjective norms are statistically associated with fertility intention, with subjective norms demonstrating a stronger association. This pattern suggests that fertility-related orientations are not only linked to individual evaluations but are also embedded within social-relational environments. As such, approaches that simultaneously address evaluative meanings (attitudes) and perceived expectations (subjective norms) may be more consistent with the observed structure of associations. For instance, community-level initiatives may create spaces for discussion among couples and families that frame fertility decisions in terms of relational harmony and mutual respect, rather than social judgment. From a culturally specific perspective, such approaches align with the role of mianzi in organizing social evaluation and identity, while remaining consistent with the observed importance of normative processes in fertility intention formation.

### 6.1 Limitations

This study has several limitations that should be acknowledged.

First, the sample is skewed toward younger and relatively highly educated women, and therefore the findings should be interpreted as capturing fertility intention formation among women in the early life-course stage with substantial educational investment, rather than realized fertility behavior across the broader population of Chinese women. Future research should extend the model to more age-diverse and socioeconomically heterogeneous samples, including married women and those with lower educational attainment, to assess the robustness of the observed relationships across different family-formation contexts.

Second, the cross-sectional research design limits the ability to draw causal inferences among the studied variables. Although the proposed relationships are theoretically grounded in the Theory of Planned Behavior and Social Identity Theory, the empirical evidence is correlational in nature. Longitudinal or panel-based studies are needed to examine how Mianzi-related concerns, attitudinal and normative processes, and fertility intentions evolve over time and to more rigorously test causal dynamics.

Third, the study’s focus on Chinese women constrains the generalizability of the findings beyond the specific cultural context examined. While Mianzi represents a salient cultural mechanism in China, similar face-related or identity-based constructs may operate differently across societies. Future research could extend this framework to other collectivist or face-oriented cultures to assess the cross-cultural applicability of the proposed model.

The last limitation of this study concerns the marital status of respondents. The survey captured marital status using a simplified categorization and did not differentiate among more detailed relationship statuses such as married, divorced, or cohabiting. As a result, potential heterogeneity in fertility intentions across distinct marital or partnership contexts could not be examined. Future research should employ more fine-grained marital status measures to better capture variation in relationship arrangements and to more precisely assess how marital and partnership dynamics relate to fertility intentions.

More important, this study adopts a theoretical specification in which mianzi is positioned as an upstream cultural–identity orientation that operates through attitudes and subjective norms within the TPB framework. While this positioning is theoretically grounded in Social Identity Theory, it remains open to debate. Given the conceptual proximity between mianzi, attitudes, and subjective norms, an alternative specification in which mianzi functions as a parallel predictor of fertility intention may also be theoretically defensible. Although model comparisons indicate that the proposed specification provides a better fit than one-factor and higher-order alternatives, such evidence does not fully resolve the underlying conceptual ambiguity. In addition, the measurement approach in this study relies on fertility-specific adaptations of mianzi items, which may partially overlap with attitudinal and normative evaluations. Future research should employ more general face-consciousness measures and explicitly compare competing model structures (e.g., mediation versus parallel models) using longitudinal or experimental designs to more rigorously establish the theoretical positioning of mianzi within the TPB framework.

## Data Availability

The original contributions presented in the study are included in the article/supplementary material, further inquiries can be directed to the corresponding author.
